# Staff risks stratification in preparation for COVID-19 in a tertiary healthcare facility in Nigeria

**DOI:** 10.11604/pamj.supp.2020.35.2.25095

**Published:** 2020-07-28

**Authors:** Darlington Ewaen Obaseki, Obehi Aituaje Akoria, Ndubuisi Mokogwu, Casimir Erhunmwun Omuemu, Benson Uchechukwu Okwara, Esohe Olivia Ogboghodo

**Affiliations:** 1 Department of Anatomic Pathology, University of Benin Teaching Hospital, PMB 1111, Benin City, Edo State, Nigeria,; 2 Department of Medicine, University of Benin Teaching Hospital, PMB 1111, Benin City, Edo State, Nigeria,; 3 Department of Community Health, University of Benin Teaching Hospital, PMB 1111, Benin City, Edo State, Nigeria

**Keywords:** COVID-19, risk stratification, healthcare workers, tertiary care hospital, Nigeria

## Abstract

**Introduction:**

this report is a documentation of a staff risk stratification programme, undertaken in University of Benin Teaching Hospital, with outcomes, and the actions taken to protect staff.

**Methods:**

an adapted risk stratification tool was circulated to all staff through their respective heads of departments/units. Staff were expected to voluntary assess their health and risk status in the context of COVID-19, using the tool. A central multi-disciplinary screening committee assessed submissions and invited staff who required further evaluation for physical interviews. Respondents were categorized into three risk/exposure groups from lowest to highest - A, B, and C, based on their individual health assessments, occupational exposures, and information obtained from direct interviews.

**Results:**

the committee received submissions from 746 staff, representing 19.4% (about a fifth) of the hospital’s 3,840 staff. One hundred and twenty two of these were invited for physical interviews, of whom 88 (72.1%) were categorized as high risk (Category C): pregnancy (53.4%); bronchial asthma (19.3%); hypertension (11.4%); cancer (3.4%) and sickle cell disease (2.3%); fractures and pulmonary tuberculosis (1%, respectively). These staff were recommended for redeployment from areas of high risk exposure to COVID-19.

**Conclusion:**

a management-driven risk assessment of hospital staff in preparation for the COVID-19 pandemic revealed that a fifth of staff assessed themselves as being vulnerable to adverse outcomes from exposure. It is our hope that similar risk stratification programmes will become standard practice in healthcare facilities during disease outbreaks, especially in Africa.

## Introduction

Healthcare workers (HCWs) are at the frontline of the SARS-CoV-2 disease outbreak response, which exposes them to the risk of infection with the pathogen which causes Corona Virus Disease, first reported in 2019 (COVID-19) [1]. The risk of exposure to SARS-CoV-2 also exists during routine duties undertaken by various categories of HCWs [2]. This risk is increased by late recognition of COVID-19 in patients, working in higher-risk departments, longer duty hours, sub-optimal adherence to Infection Prevention and Control (IPC) measures and lack of, or improper use of personal protective equipment (PPE) [3,4]. The World Health Organization (WHO) posits that employers and managers in health facilities must ensure that all necessary preventive and protective measures are taken to minimize health risks, in addition to making available adequate IPC and PPE supplies in sufficient quantity to healthcare and other staff [1]. Early in June 2020, 230,000 cases of COVID-19 had been reported among HCWs globally [5]. Data from the United Kingdom show that 13.8% of confirmed COVID-19 cases were key personnel in the National Health Service [6]. In the United States, 92,572 cases of COVID-19 had been reported among HCW with 507 deaths as of July 2020 [7]. The International Council of Nurses reported 600 deaths from COVID-19 amongst nurses, globally [5].

In China, more than 3,300 healthcare workers were infected [6]. Fifty thousand healthcare professionals have been infected in Spain - representing 20% of all recorded cases, with 70 deaths [8]. Nigerian HCWs have been affected, with over eight hundred reported to have been infected with SARS-CoV-2 by June 2020 [9]. The increasing incidence of COVID-19 in HCWs impacts negatively on hospital services, healthcare delivery, and the risk of community transmission of the disease. Uninfected workers suffer longer working hours, fatigue, and occupational burnout from the increased workload [3]. Furthermore, asymptomatic infected HCWs pose additional risks of infection to colleagues, family members and other close contacts [10]. Healthcare staff and others who work with patients during this pandemic are also exposed to mental health risks arising from exposure (or fear of exposure) to the virus, concerns about availability and adequacy of IPC commodities, as well as concerns for their personal safety [11]. COVID-19 is more severe in persons older than 60 years, those with pre-existing diseases, e.g., cardiovascular diseases, diabetes mellitus, and disorders associated with reduced immunity, and in pregnant women [12-14].

Mitigating the risks of exposure of health workers should, therefore, be a deliberate focus of healthcare managers. Risk assessment tools are useful in recognizing the magnitude of exposure among HCWs, defining the extent of transmission within healthcare facilities, and finding out the best approaches to safeguarding HCWs against infection [3]. However, proactively excluding persons in the health workforce who are judged to be at higher risk of severe disease and death from COVID-19 is recommended. Such actions would require a workforce risk assessment to identify persons with potentially increased risks of infection or adverse outcomes from COVID-19 [12]. The first cases of COVID-19 in Nigeria and Edo State (where the University of Benin Teaching Hospital is located) were reported on February 27, and March 23, 2020, respectively [15,16]. Following the report of the index case in Nigeria, the Management of UBTH had on March 23, 2020, developed a staff risk assessment tool and launched a programme to assess each worker’s risk of exposure to COVID-19 concerning their work schedules. The goal was to ensure that appropriate precautionary measures were taken to protect staff based on the guidance that was provided. The risk assessment programme was before the first case of COVID-19 was reported in the hospital on March 30, 2020. Our review aims to document the risk stratification processes, its outcomes, and the actions that were taken.

## Methods

**Setting:** the University of Benin Teaching Hospital is an 850-bed tertiary hospital with outpost facilities, six training schools, 29 clinical departments, and 35 non-clinical departments. It is located in Benin City, Edo State, Nigeria. The hospital provides specialized and multi-disciplinary healthcare services through several clinical departments [17].

**Design:** this risk assessment programme was initiated by the management of University of Benin Teaching Hospital (UBTH), in response to the announcement of index cases of COVID-19 in Nigeria, and in Edo State, within a space of almost four week (February 27 and March 23, respectively). The UBTH management, in anticipation of COVID-19 cases presenting to the hospital, made concerted efforts to ensure that staff with increased vulnerabilities to severe COVID-19, with the possibility of fatal outcomes, were exempted from the front lines.

**Figure 1:**
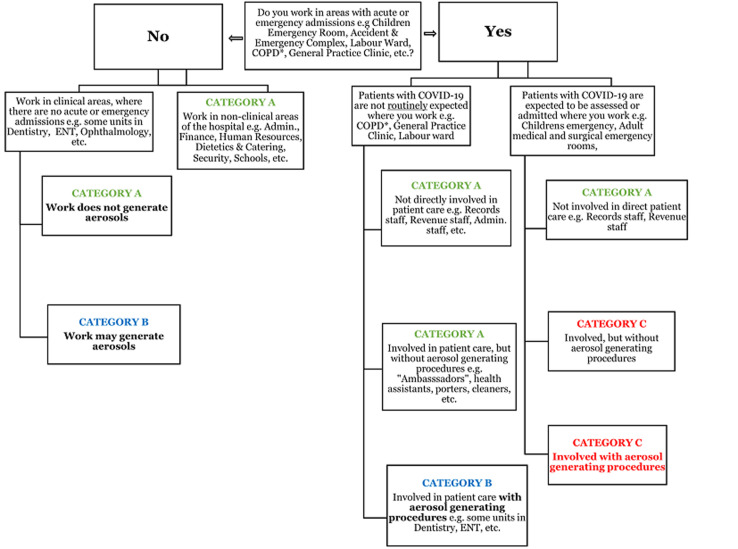
University of Benin Teaching Hospital staff risk assessment & guidance for COVID-19

**Development of the risk assessment tool and staff sensitization:** a team of 3 senior doctors adapted the risk stratification tool from the “Individual Staff Risk Assessment Checklist for Covid-19” published by the NHS East and North Essex Foundation Trust (ESNEFT) Occupational Health and COVID-19 IMT [18] (Figure 1). The adapted tool was presented to management staff and heads of departments across the hospital on March 24, 2020. An internal memorandum was subsequently circulated from management to staff across 71 clinical and non-clinical departments, units, and offices in the hospital, informing them of the forthcoming risk assessment programme and requesting their cooperation. The tool included sections on: (1) Individual Health Assessment; (2) Occupational Exposure in Patient Areas; and (3) Exposure and Risk Categories, with guidance on actions to be taken.

**Risk assessment procedure:** all heads of departments received printed copies of the risk assessment tool and were mandated to disseminate these to all members of staff in their respective departments. Staff were required to indicate if their routine duties involved regular work in patient areas or regular patient contact. If a staff worked in areas with regular patient contact, they were further categorized based on whether they worked in acute or emergency admission areas; if there could be contact with suspected COVID-19 cases, and/or exposure to aerosol-generating procedures (AGPs). Individual staff voluntarily completed the risk assessment checklists, which were submitted to the Central Risk Assessment Screening Committee through the respective department heads. The screening committee comprised management staff, with senior clinical, and non-clinical staff of the hospital.

**Risk categorization:** a priori, high-risk staff included persons with chronic diseases such as hypertension, diabetes mellitus; lung, cardiovascular and kidney diseases; neurological disease and immunosuppression. Persons on regular treatment with immunosuppressive medicines and pregnant women were also considered high risk. Each staff was categorized into Categories A, B, and C based on their considered occupational exposure risk (Figure 1). The risk assessment screening committee reviewed submissions and invited members of staff to appear physically for further screening. Staff were subsequently categorized into three risk/exposure groups, from lowest (Category A) to highest (Category C) based on a global assessment of individual health, occupational exposures, and further information obtained from direct interaction.

## Results

Risk assessment checklists for 746 staff, from 24 departments and units were submitted to the screening committee. This represents voluntary participation of 19.4% of the 3,840 staff of the hospital. Of these, 122 (16.4%) were invited for further physical screening by the screening committee. Eighty-eight (72.1%) of these (i.e. 11.8% of all staff who voluntarily participated) were categorized as high risk (Category C). This staffs were thus recommended for exclusion from areas of high risk exposure to COVID-19. Majority of staff in Category C were from the Nursing Services department, and female (96.6%) - Table 1 and Table 2, respectively. Clinical staff were the majority (87.5%) of healthcare workers who were granted exemptions: nurses (56.8%) and medical doctors (18.2%) made up three-quarters of staff in this category. Administrative staff working in clinical areas made up 8.0%, while laboratory scientists/technicians made up 6.8% (Table 2). The criteria for placing staff in Category C included pregnancy (53.4%); bronchial asthma (19.3%); hypertension (11.4%); cancer (3.4%) and sickle cell disease (2.3%). Other conditions were fractures and pulmonary tuberculosis (Table 3).

**Table 1:** staff risk stratification by department/unit

S/No.	Department/Unit	Total no. of staff	No. of staff screened in person	No. of staff in Categories A and B	No. of staff in Category C (high risk/exemptions)
1.	Nursing Services	776	60	10	50
2.	Family Medicine	56	5	1	4
3.	National Health Insurance Scheme	40	8	4	4
4.	Chemical Pathology	54	3	0	3
5.	Internal Medicine	62	3	1	2
6.	Medical Microbiology	90	4	2	2
7.	Medical Records	140	4	2	2
8.	Medical Social Services	24	2	0	2
9.	Obstetrics & Gynaecology	71	2	0	2
10.	Ophthalmology	47	2	0	2
11.	Post Basic School of Nursing	46	4	2	2
12.	Radiology	98	2	0	2
13.	Restorative Dentistry	25	2	0	2
14.	SERVICOM	16	3	1	2
15.	Anaesthesia	113	3	2	1
16.	Haematology	69	2	0	2
17.	Occupational Therapy	39	1	0	1
18.	Oral & Maxillofacial Surgery	43	1	0	1
19.	Periodontics	11	1	0	1
20.	Preventive Dentistry	27	1	0	1
21.	Human Resources	107	6	6	0
22.	Institute of Health Technology	76	1	1	0
23.	Office of the Chief Medical Director	44	1	1	0
24.	Procurement	28	1	1	0
25.	Total	2,102	122	34	88

**Table 2:** characteristics of staff categorized for exemptions (category C[PDO1] )

Characteristics	Frequency (n =88)	Percent
**Sex**		
Female	85	96.6
Male	3	3.4
**Staff category**		
Clinical	77	87.5
Non-clinical	11	12.5
**Professional category**		
Nursing	50	56.8
Medical	16	18.2
Administration	7	8.0
Laboratory	6	6.8
Nurse tutoring	2	2.3
Medical records	2	2.3
Medical Social Services	2	2.3
Radiography	1	1.1
Occupational therapy	1	1.1
Support staff	1	1.1
		

**Table 3:** reasons for which staff were given exemptions

Reasons for exemptions	Frequency (n=88)	Percent
Pregnancy	47	53.4
Bronchial asthma	17	19.3
Poorly controlled hypertension	10	11.4
Hypertension with diabetes mellitus	4	4.6
Diabetes mellitus	3	3.4
Cancer	3	3.4
Sickle cell disease	2	2.3
Fracture	1	1.1
Pulmonary tuberculosis	1	1.1

Actions based on staff risk stratification: staff in Category A were adjudged to be able to remain in their current roles or in other roles with similar duties, and were so advised. Staff in Category B were similarly adjudged, but were further instructed to ensure adherence to standard infection prevention control measures, and to use personal protective measures appropriately. Staff in Category C were redeployed to other work areas with less risks of exposure to patients with COVID-19, or requested to stay away from work temporarily. The respective heads of departments determined appropriate workplace adjustments based on the occupational risk categories of individual staff, and communicated these adjustments to hospital management. All staff who were temporarily asked to stay away from work continued to earn their full pays. Periods of exemption from usual duty depended on the nature of the risk and the physical/health status of the staff. For example, persons with chronic medical conditions were redeployed to areas with less risk of exposure to suspected or confirmed cases of COVID-19, while those who were in late stages of pregnancy were advised to stay away until after delivery.

## Discussion

This report highlights the processes and outcomes of a risk stratification programme undertaken by the management of University of Benin Teaching Hospital, Nigeria in response to the COVID-19 pandemic. It may nearly be impossible to eliminate risks of exposure to disease-causing pathogens in the workplace, but employees must be given a level of protection that is commensurate with their individual risks and exposure levels. This is the core of Legges’ aphorism which states that every employer is duty bound to safeguard the health of workers under their employment [19]. This is a principle that should be adhered to, if we are to have a healthy and efficient workforce. There are several benefits of workplace risk assessment programmes. Risk stratification during such programmes enables the development of preventive guidelines to protect healthcare workers with unequal vulnerability to a given disease. They could also potentially decrease health disparities [20]. In the context of COVID-19, staff risk assessments can help to ensure that suitable adjustments are made to reduce healthcare workers´ risks of contracting the disease - this is especially important for high-risk staff.

All staff members were expected to voluntarily participate in the risk stratification exercise, but only about 20% submitted their individual risk assessment documentation. This may suggest that the majority of staff in the hospital considered themselves healthy enough, and were willing to participate in the COVID-19 “fight”. We believe that undertaking this exercise would have gendered a sense of being valued and protected amongst staff, particularly as staff who were adjudged to be at high risk were redeployed to safer working conditions. A critical feature of the current COVID-19 pandemic is the number of healthcare workers who have been infected, and who have succumbed to the disease. In the United States, more than ninety thousand cases of COVID-19 had been reported among healthcare workers, with over five hundred deaths, as of July 2020 [7]. This situation is particularly worrisome when we consider how much time and resources are required to train and certify healthcare workers. The implications are particularly worrisome for health systems in developing countries in Africa where lack of investment in healthcare infrastructure and poor working conditions are making it increasingly hard to retain skilled healthcare workers [21,22].

Africa suffers more than 22% of the global burden of disease but has access to only 3% of healthcare workers, and less than 1% of the world’s financial resources [23]. In resource-constrained settings in Africa therefore, it behooves healthcare leaders and managers to protect the ever-shrinking healthcare workforce. According to WHO, a minimum of 23 doctors, nurses and midwives per 10,000 population are required to deliver essential maternal and child health services [24]. Sadly, Nigeria falls below this minimum requirement, with only 20 medical doctors, nurses and midwives per 10,000 population [24]. The challenges associated with inadequate numbers of healthcare staff are compounded in pandemic situations, and would be further worsened by healthcare worker infections if allowed to happen without proactive interventions. The death of one healthcare worker as a result of COVID-19 would no doubt cause shocks to the entire healthcare system, and create gaps in service delivery [23], not only from the direct effects of such loss, but also from the myriad of indirect effects. Health facility managers need to take into consideration their local settings and other factors when making decisions following risk stratification [12]. These decisions should be made in consultation with relevant staff [12]. As highlighted earlier, the management of University of Benin Teaching Hospital worked with department and unit heads, and individual staff, to identify persons with increased vulnerability to COVID-19 and redeployed them from work places with high risks of exposure to other work areas.

Researchers in the United Kingdom reported that age above 70 years, male sex, underlying health conditions, pregnancy and certain ethnic groups such as black and minority ethnic health populations increase vulnerability to adverse outcomes of COVID-19 [12]. Documentation from our staff risk stratification indicated that no staff was exempted due to age. This is not surprising, considering that retirement age is statutorily 60 years, There is however a small proportion of staff who are aged 60 years and above (university lecturers), who may on their own have assessed their high risk status and chosen not to participate in the hospital risk assessment. Core clinical staff made up more than four-fifths of workers in the high risk category. This again, should not be surprising, nor should the finding that nurses and medical doctors made up three-quarters of this group: the risk assessment was for vulnerability to COVID-19, clinical staff on the front lines are more likely to be exposed. The non-clinical staff who were adjudged to be at high risk were those who worked in clinical areas. It would have been useful to provide information on how the absence of staff who were redeployed from high risk areas affected the running of their units. This is however beyond the scope of this report.

## Conclusion

Our staff risk assessment programme was aimed at identifying and protecting health workers with higher vulnerability to the adverse effects of COVID-19. It revealed that about 20% of staff considered themselves to be at some risk, of whom 16% were categorized as high risk, and subsequently redeployed. It is our hope that such risk stratification programmes will become standard practice in healthcare facilities in similar settings, especially in Africa, during disease outbreaks.

### What is known about this topic

- Risk stratification enables the development of preventive guidelines which will protect healthcare workers who are vulnerable to a given disease.

### What this study adds

- Highlights proactive measures that were directed at mitigating the effects of COVID-19 in vulnerable members of the healthcare workforce;
- Managers of healthcare facilities in similar settings may glean lessons which could be applied to safeguard the health and wellbeing of their staff;
- Furthermore, this risk stratification exercise underscored the value of team work and collaboration in healthcare administration.
